# The Advanced Therapeutics for Alzheimer’s Disease Review Board: A Multidisciplinary Model to Facilitate Safe Access to Lecenemab across the Sutter Health System

**DOI:** 10.1002/alz.093375

**Published:** 2025-01-09

**Authors:** Armen Moughamian, Shawn Kile, Travis Urban, Chen Zhao, Michelle Manifield, Jean Minton

**Affiliations:** ^1^ Ray Dolby Brain Health Center, Sutter Health, San Francisco, CA USA; ^2^ Sacramento Memory Center, Sutter Health, Sacramento, CA USA; ^3^ Sutter Health Neuroscience Service Line, Sacramento, CA USA

## Abstract

The full approval by the FDA of the first disease‐modifying therapy for Alzheimer’s disease has significantly impacted the treatment paradigm of the disease. Safe administration of lecanemab requires a greater level of care and monitoring and increases the care‐burden on patients, their care‐team, and the health system. Prior to the approval of lecanemab, a significant shortage of cognitive specialists already existed. The Sutter Health system treats over 3 million patients across Northern California but has only 4 cognitive neurology specialists. In the face of a limited number of cognitive neurologists, we set out to develop a system‐wide approach to improve access to advances in the treatment of Alzheimer’s disease. The Advanced Therapeutics for Alzheimer’s Disease Review Board emerges as an innovative model to promote the safe, equitable, efficient and local access to these novel therapies. The board is comprised of a multidisciplinary team of specialists, including cognitive neurologists, a neuroradiologist, a pharmacist, and an RN navigator. General neurologists throughout the Sutter Health system submit cases to the board which meets weekly. The board plays a crucial role in reviewing and guiding the local practitioners in the appropriate use for patients considering treatment. The board enables local neurologists to safely prescribe lecanemab and improves access to these advanced therapeutics in diverse and underserved populations. The board further enhances safety by re‐reviewing cases in which local providers have concerns about the management of side‐effects, including as ARIA. Outcomes of our innovative model including access to and safety of treatment will be tracked. Lastly, as additional advanced treatment options for Alzheimer’s disease are approved, we anticipate that the Advanced Therapeutics of Alzheimer’s Disease Review Board will be able to assist general neurologists in the Sutter Health network with the appropriate selection of these novel agents. Sutter Health’s Advanced Therapeutics for Alzheimer’s Disease Review board is a novel system‐wide model to address the shortage of cognitive specialists while enhancing the safe and equitable access to advanced treatments for Alzheimer’s Disease across a health system.

